# Mortality following adolescent substance treatment: 21-year follow-up from a single clinical site

**DOI:** 10.3389/frcha.2025.1600101

**Published:** 2025-06-20

**Authors:** Christian Thurstone, Cassandra Etzig, Eileen Chen, Hayley D. Seely, Ryan Loh

**Affiliations:** ^1^ Behavioral Health Services, Denver Health and Hospital Authority, Denver, CO, United States; ^2^ Department of Psychiatry, University of Colorado School of Medicine, Aurora, CO, United States

**Keywords:** adolescent, mental health, mortality, substance misuse, treatment

## Abstract

**Background:**

Despite known risks of substance use, mortality following adolescent substance treatment has not been examined. Knowing which youth have greatest risk and how youth die may inform future interventions.

**Methods:**

This retrospective chart review combined records from a single adolescent substance treatment program at an urban, safety-net health system (*n* = 2,957, ages 10–19 years) with a public health registry of deaths from 2003 to 2024. Records from the child mental health treatment program at the same health system (*n* = 4,400, ages 10–19 years) were used for comparison. The crude mortality rate per 100 person years was calculated for both samples for all 21 years. The standard mortality rate using death within one year of intake was also calculated. Finally, a logistic regression model was used to test the project hypotheses that self-identification as a person of color, no engagement in care, male sex at birth, and involvement in substance treatment would predict mortality.

**Results:**

Overall, 92 (2.1%) adolescents in mental health treatment had a death record compared to 119 (4.0%) of adolescents in substance treatment. The crude mortality rate per 100 person years for adolescents in mental health treatment was 0.19 (CI: 0.18–0.20) compared to 0.37 (CI: 0.36–0.38) for adolescents in substance treatment. The standard mortality rate was 120 (32.7–308) for adolescents in mental health treatment compared to the standardized mortality rate of 944 (CI: 599–1,420) for adolescents in substance treatment. Accidental death, which includes overdose, was the most common cause of death in both groups. Results of the logistic regression showed male sex at birth (*p* = 0.0434, OR = 2.10, CI 1.06–4.53) and substance treatment (*p* = 0.0035, OR = 3.02, CI 1.47–6.55) as predictors of death within 5 years of treatment intake.

**Conclusions:**

Adolescents in substance treatment compared to those in mental health treatment are more likely to die within 5 years of intake. Males compared to females are also more likely to die. Interventions to prevent overdose and other causes of mortality may be indicated.

## Introduction

Substance use disorders (SUD) and psychiatric disorders are the 18th and 22nd leading causes of global death, respectively (1). In the European Union, the mortality rate due to overdose alone is 22.5 deaths per million population aged 15–64 (2). In the United States, three different substances (tobacco, alcohol, and illegal) account for three of the top 10 leading causes of death (3). As a result, death due to these disorders is common and widespread. Consistent with these findings, adolescents seeking psychiatric treatment have elevated risk of mortality. For instance, a study of almost 60,000 adolescents with an inpatient psychiatric admission in the United Kingdom shows a standard mortality rate of 642 (CI: 532–767), with 100 being a reference standard mortality rate (4). Standard mortality is typically defined as the observed number of deaths in a sample divided by the expected number of deaths in a population of similar age and sex multiplied by 100 (4). Therefore, rates greater than 100 indicate greater than average risk of death, and rates less than 100 indicate a lower risk of death compared to the general population. Another sample in Sweden shows that adolescents with either an inpatient or outpatient mental health encounter had a standard mortality rate of 221 (CI: 156–303) (5). Mortality risk factors for adolescents entering mental health treatment include affective disorders, co-occurring substance use, eating disorders, history of psychosis, older age at intake, and school problems (4, 6, 7). Conversely, having an outpatient appointment within 7 days of discharge from an adolescent psychiatric hospital is a protective factor against mortality (8).

Among adolescents, substance use is associated with the leading causes of death, which include accidents and injuries, self-harm, and interpersonal violence (9). Adolescent substance use is also complicated by a high prevalence of co-occurring psychiatric disorders (Dennis et al., 2004) (10). Furthermore, substances such as cannabis are strongly associated with psychiatric symptoms such as psychosis (11–13). Despite the devastating outcomes of adolescent substance use and the known risk of mortality for adolescents in psychiatric treatment more generally, mortality following adolescent substance treatment remains understudied. Most studies focus on adults seeking substance treatment and show an elevated risk of mortality compared to the general population (14, 15). Mortality risk factors for adults in substance treatment include chronic medical illness, homelessness, older age at intake, past suicide attempt, male sex, and alcohol or heroin as a primary substance use disorder diagnosis (16–19). Moreover, for adults in behavioral health treatment, substance use disorders are associated with increased mortality compared to other psychiatric disorders (20). Additionally, in the United States age adjusted mortality from substance use disorders increased significantly from 2000 to 2019, especially for African American and American Indian/Alaska Native populations (14). These groups now experience the highest mortality rates from substance use disorders compared to other racial and ethnic groups (14).

While adolescents in mental health treatment and adults in substance treatment both face elevated risks of mortality, there is a lack of research documenting mortality for adolescents engaging in substance treatment. Investigating this area is important, as it could inform interventions to prevent mortality in this population. Extrapolating from the literature reviewed above, we hypothesize that adolescents in substance treatment have elevated risk of mortality compared to adolescents in mental health treatment and that risk factors for mortality include older age at intake, male sex, and having an ethnic or racial identity with a history of marginalization. We also hypothesize that successful treatment engagement is protective. The objective of this project is to evaluate these hypotheses using data from an adolescent substance treatment program with a 21-year follow-up.

## Materials and methods

### Overview

This project is a retrospective chart review, which explores mortality following referral to adolescent mental health and substance treatment.

### Procedure

All adolescent patients (10–19 years of age) presenting to an urban, safety-net health system in Denver, Colorado, for either mental health or substance treatment between January 2, 2003, and July 8, 2024, were included in the analysis. Data were extracted from the electronic health record for analysis. These data were cross-referenced with a public health database of mortality. The Revised Standards for Quality Improvement Reporting Excellence (SQUIRE 2.0) are used to carry out the project and report its findings (21). The project was reviewed by the Colorado Multiple Institutional Review Board and was determined to be non-human subjects research.

### Intervention

All adolescents had at least one appointment in either a state-licensed mental health or substance treatment program. Adolescents who received treatment in both programs were placed in the adolescent substance treatment program to avoid duplication. Treatment included outpatient psychotherapy and/or medication management depending on clinical indication and patient preference.

### Measures

Baseline demographic variables were extracted from the electronic health record. These include age at intake, ethnicity, race, sex at birth, and whether adolescents had successful treatment engagement, which is defined by the Healthcare Effectiveness Data and Information Set (HEDIS) as having two or more treatment sessions within 34 days of intake (22). Mortality data included death as a binary variable (yes/no), date of death, and manner of death. Manner of death included the six categories of accidental, homicide, natural, pending, and undetermined. Of note, unintentional overdose is typically categorized as accidental.

### Data analysis

Data were analyzed using RStudio (V.2024.04.01) and tidyverse (23). Descriptive statistics are used to characterize the sample. To compare to previous research, a crude mortality rate per 100 person years was calculated for each sample (16). Crude mortality rate is calculated by dividing the number of deaths in the sample by the sum of the years each person in the sample was followed and multiplying by 100 (16). Assuming a Poisson distribution for crude mortality rate, a 95% Confidence Interval (CI) was also calculated. A standardized mortality rate was calculated by dividing observed deaths by expected deaths and multiplying by 100. Expected deaths were calculated by age group (10–14-year-olds, 15–19-year-olds) and sex using published mortality rates for the United States (24). Confidence intervals were calculated assuming Poisson distributions for standard mortality. For formal analyses, ethnicity and race were categorized dichotomously as Black, Indigenous, or Person of Color (BIPOC) or Non-Hispanic White. This dichotomization was done because of shifting standards in the collection of data related to ethnicity and race since 2003 and because of a small sample size of some races. Similarly, sex at birth was categorized as a binary variable (female, male) because of changing standards of reporting sex and gender since 2003. A logistic regression model was used to test the project hypotheses identification as a person of color, no engagement in care, male sex at birth, and involvement in substance treatment would predict mortality. For the logistic regression, the binary outcome of death within 5 years of intake (yes, no) was used as the dependent variable. Therefore, this model used a sub-sample of individuals who sought treatment at least five years before the dataset was created (July 8, 2024). This standardization was chosen to provide meaningful comparison between adolescents in the two programs and to assess outcomes temporally related to treatment.

## Results

The sample included 4,400 adolescents in mental health treatment and 2,957 adolescents in substance treatment. Table 1 displays the baseline characteristics of the sample. Generally, adolescents in substance treatment were slightly older and were more likely to have male sex at birth compared to adolescents in mental health treatment. The most common substance use disorder diagnoses were cannabis use disorder (*n* = 2,096, 70.9%, alcohol use disorder (*n* = −665, 22.5%), stimulant use disorder (*n* = 424, 14.3%), and opioid use disorder (*n* = 366, 12.4%). Overall, 92 (2.1%) adolescents in mental health treatment had a death record compared to 119 (4.0%) of adolescents in substance treatment. The crude mortality rate for adolescents in mental health treatment was 0.19 (CI: 0.18–0.20) per 100 person years compared to 0.37 (CI: 0.36–0.38) per 100 person years for adolescents in substance treatment. The standardized mortality rate for adolescents in substance treatment was 120 (32.7–308) for adolescents in mental health treatment compared to 944 (CI: 599–1,420) for adolescents in substance treatment. The manner of death is displayed in Table 2. Accidental death was the most common cause of death in both groups but appears to be slightly more prevalent among adolescents in substance treatment. Table 3 compares deaths by ethnicity, race, and sex at birth. For both groups, death appears to be more common among males.

**Table 1:** Baseline description of the sample.

Variable	Mental health treatment (*n* = 4,400)	Substance treatment (*n* = 2,957)
Age, mean (SD)	14.0 (2.43) years	15.7 (1.71) years
Ethnicity—*n* (%)		
Hispanic/Latinx	2,157 (49.0%)	1,319 (44.6%)
Not Hispanic/Latinx	2,186 (49.7%)	1,594 (53.9%)
Other/Declined	57 (1.3%)	44 (1.5%)
Race—*n* (%)		
American Indian or Alaska Native	62 (1.4%)	43 (1.5%)
Asian	64 (1.5%)	34 (1.1%)
Black or African American	665 (15.1%)	399 (13.5%)
Native Hawaiian or Other Pacific Islander	7 (0.16%)	6 (0.20%)
White	2,803 (63.7%)	517 (17.5%)
Other or declined	799 (18.2%)	517 (17.5%)
Sex at birth—*n* (%)		
Female	2,359 (53.6%)	967 (32.7%)
Male	2,041 (46.4%)	1,990 (67.3%)

**Table 2:** Manner of death.

Manner—*n* (%)	Mental health treatment (*n* = 92 deaths)	Substance treatment (*n* = 119 deaths)
Accidental	28 (30.4%)	53 (44.5%)
Suicide	22 (23.9%)	22 (18.5%)
Natural	20 (21.7%)	15 (12.6%)
Homicide	16 (17.4%)	11 (9.2%)
Undetermined	3 (3.3%)	3 (2.5%)
Pending	3 (3.3%)	15 (12.6%)

**Table 3:** Description of deaths by ethnicity, race, and sex at birth.

Variable—*n* (%)	Mental health treatment (92 deaths)	Substance treatment (*n* = 119)
Ethnicity		
Hispanic/Latinx	34 (37.0%)	47 (39.5%)
Not Hispanic/Latinx	56 (60.9%)	70 (58.8%)
Other or decline	2 (2.2%)	2 (1.7%)
Race		
American Indian or Alaska Native	0 (0%)	2 (1.7%)
Asian	0 (0%)	1 (0.84%)
Black or African American	14 (15.2%)	15 (12.6%)
Native Hawaiian or Other Pacific Islander	0 (0%)	0 (0%)
White	53 (57.6%)	77 (64.7%)
Other or declined	25 (27.2%)	24 (20.2%)
Sex at birth		
Female	30 (32.6%)	30 (25.2%)
Male	62 (67.4%)	89 (74.8%)

The project hypotheses were tested with a logistic regression using the independent variables of age, engagement in care, dichotomization of race and ethnicity as BIPOC or Non-Hispanic/White, sex at birth, and whether the adolescent was in mental health or substance treatment. This analysis included a sub-sample for whom 5-year follow-up data were available (*n* = 3,188 in mental health treatment and *n* = 2,331 in substance treatment). Twelve adolescents (0.38%) in mental health treatment died within 5 years of intake, and 32 adolescents in substance treatment died within 5 years of intake. Results of the logistic regression showed male sex at birth (*p* = 0.0434, OR = 2.10, CI 1.06–4.53) and substance treatment (*p* = 0.0035, OR = 3.02, CI 1.47–6.55) as predictors of death within 5 years of treatment intake. Age at intake, engagement in care, and race/ethnicity were not statistically associated with death within 5 years.

## Discussion

This retrospective review finds that 0.38% of adolescents in mental health treatment and 1.4% of adolescents in substance treatment died within 5 years of intake. Having male sex and enrollment in substance treatment were significant predictors of mortality. This project did not find ethnicity, race, or treatment engagement as significant predictors of death. The most common cause of death was accidental, including unintentional overdose, followed by suicide.

The study has several limitations. First, its use of a single site limits the generalizability of the findings. Future research is needed to evaluate our findings in other geographical locations. Second, as a retrospective review, the study is susceptible to potential confounds. Finally, standards for collecting demographic variables, such as ethnicity, race, and sex have evolved since the data collection began in 2003.

In context, these data show a crude mortality rate of 0.37 per 100 person years for adolescents in substance treatment compared to crude mortality rates ranging from 0.30 to 1.48 per 100 person years (depending on primary substance diagnosis) for a national sample of all patients entering substance treatment in New Zealand (16). Our data show a standardized mortality rate of 944 for adolescents in substance treatment compared to previous findings of a standard mortality rate of 642 for adolescents with an inpatient psychiatric admission and 221 for adolescents with either an inpatient or outpatient mental health encounter (4). Our finding that males compared to females have greater mortality risk is also consistent with previous findings in other populations in substance treatment (17). Therefore, our data are generally consistent with previous reports and suggest that adolescents in substance treatment carry increased mortality risk compared to adolescents in treatment for other psychiatric disorders. However, our finding that ethnicity and race did not predict mortality is not consistent with previously documented disparities (14). Perhaps, the shifting effects by ethnicity and race of the different waves of the opioid overdose epidemic account for this inconsistency (25).

These data are important because they suggest that adolescents in substance treatment may benefit from interventions to reduce their mortality. Given that the leading cause of death was accidental, which includes overdose, harm reduction approaches such as encouraging adolescents to use designated drivers, carry naloxone, use fentanyl testing strips, and avoid using substances alone may be beneficial. Since suicide and homicide were also significant contributors to death, interventions that address gun violence could be helpful. Using Learning Health System approaches to implement and sustain evidence-based suicide interventions into substance treatment such as the Collaborative Assessment and Management of Suicidality (CAMS) could also be an important future direction (26). Finally, adolescent males in substance treatment may benefit from targeted screening and interventions aimed at reducing mortality. Future directions could include an evaluation of our findings in different locations and with different populations to inform future population-based prevention efforts.

## Data Availability

The original contributions presented in the study are included in the article/Supplementary Material, further inquiries can be directed to the corresponding author.
