# Correction: Mortality following adolescent substance treatment: 21-year follow-up from a single clinical site

**DOI:** 10.3389/frcha.2025.1656174

**Published:** 2025-07-14

**Authors:** Christian Thurstone, Cassandra Etzig, Eileen Chen, Hayley D. Seely, Ryan Loh

**Affiliations:** 1 Behavioral Health Services, Denver Health and Hospital Authority, Denver, CO, United States; 2 Department of Psychiatry, University of Colorado School of Medicine, Aurora, CO, United States

**Keywords:** adolescent, mental health, mortality, substance misuse, treatment

In the published article, an author name was incorrectly written as Haley D. Seely. The correct spelling is Hayley D. Seely.

The authors apologize for this error and state that this does not change the scientific conclusions of the article in any way. The original article has been updated.

